# Aquaculture Feeds Can Be Outlaws for Eutrophication When Hidden in Rice Fields? A Case Study in Qianjiang, China

**DOI:** 10.3390/ijerph16224471

**Published:** 2019-11-13

**Authors:** Chunhui Liu, Naijuan Hu, Weixuan Song, Qian Chen, Liqun Zhu

**Affiliations:** 1 College of Humanities & Social Development, Nanjing Agricultural University, Nanjing 210095, China; lch@njau.edu.cn (C.L.); hunaijuan@njau.edu.cn (N.H.); 2018810098@njau.edu.cn (Q.C.); 2 Institute of Regional Agricultural Research, Nanjing Agricultural University, Nanjing 210095, China; 3 Nanjing Institute of Geography and Limnology, Chinese Academy of Sciences, Nanjing 210008, China; wxsong@niglas.ac.cn; 4 Key Laboratory of Watershed Geographic Sciences, Chinese Academy of Sciences, Nanjing 210008, China

**Keywords:** water eutrophication, environmental governance, integrated agriculture–aquaculture systems, crayfish and rice integrated system

## Abstract

Water eutrophication caused by agricultural production has become one of the most important factors that impede sustainable rural environmental governance in China. As a result, the Chinese central and local governments want to reduce the use of nitrogen and phosphorus fertilizer and gain socioeconomical profit simultaneously by promoting crayfish and rice integrated system (CRIS) in the rural areas with abundant water resources. In this article, we investigated whether CRIS in Qianjiang, Hubei, the origin place of the system in China, contributes to fulfilling the governments’ expectations. We found that CRIS efficaciously cuts the fertilizer rate in rice production and boosts farmers’ incomes because crayfish has a demand for water quality and holds a large internal market requirement. However, higher profit encourages farmers to expand crayfish production and thus discourages the initiatives in rice production. The area of the ditch for crayfish production expands ceaselessly and exceeds the limit of regulation of CRIS. As a result, the CRIS in the areas has emerged as a practice of aquaculture but in farmland. This is a regulatory gap. The input–output analysis of CRIS by material balance method can also reveal that excessive feed for crayfish has become a new source of agricultural pollution. Beyond that, due to the changed irrigation system and increased water exchange frequency of CRIS, the pollution has transformed from passive distribution to active, which will increase the risk of water eutrophication on a large area.

## 1. Introduction

Excessive nitrogen (N) and phosphorus (P) can result in eutrophication due to water enrichment with reactive N and P for excessive algal and other aquatic plant growth [1]. More seriously, N and P can spread to a broader range by water cycle system. Thus, in most cases, eutrophication is caused by non-point sources and is hard and expensive to control. Although N and P can derive from a variety of sources, nutrient fertilizers and animal waste are the primary sources of excessive N and P. As a result, rural areas and developing countries are especially affected by this environmental problem with their agricultural activities [2,3], and China is no exception. According to statistics, from 2005 to 2016, fertilizer application in plant production increased from 26 million tons to 60 million tons in China [4]. However, the effects of these loads of nitrogen and phosphorous extend beyond the field and past the growing season. Only 35% of the N and P can be absorbed by plants, while the rest finally ends up in rivers, lakes, and oceans [5,6]. Thus, agriculture has been the most significant contributor for both N and P emissions (e.g., 57.2% and 67.4% emissions of N and P, respectively), and has displaced industry as the primary source of water pollution in China [7,8].

Due to increasing deteriorating water quality associated with eutrophication, a variety of strategies have been suggested to control the pollution, including (1) transfer of excess N and P [9], (2) changing nutrient ratios [10], (3) physical mixing [11], and (4) application of potent algaecides and herbicides [3]. However, because of the complexity and hugeness of ecosystems, these strategies had less success in dealing with the problem [3]. A more effective way to control the water eutrophication is to reduce N and P input into water cycle system [12,13]. Because integrated farming can diminish agroindustry inputs by recycling the agricultural byproduct, it is widely accepted that integrated farming can reduce the use of pesticides and fertilizers to limit the diffusion of N and P [14]. Thus, integrated farming, such as integrated agriculture–aquaculture systems (IAAS) as well as recirculating aquaculture systems, has been practiced in many countries [15,16].

China has a long history of integrated farming, especially IAAS [17]. Up to 2017, the IAAS area in China has reached 1.87 million hectares, and the trend is increasing [18]. Along with a booming crayfish market, crayfish and rice integrated system (CRIS), a typical example of IAAS, is taking up a more significant share of China’s IAAS production area in recent years. Chinese center and local governments hope to promote CRIS in the rural areas with abundant water resources for reducing the use of N and P fertilizer and gaining socioeconomical profit simultaneously. As pesticides and fertilizer adversely affect crayfish production, CRIS efficaciously cuts the pesticides and fertilizer rate in rice production. Recent research found that CRIS largely achieved the goals that the governments set when seeking a way to increase rural incomes and reduce the use of pesticides and fertilizer [19,20,21]. However, most studies have focused solely on the economic benefits of crayfish. Moreover, recent studies on the ecological benefits of CRIS and IAAS focused on its benefits for rice production, which is just a part of the system [22,23,24]. There is a concern about whether crayfish production of CRIS presents new problems for the rural ecological environment. Under this scenario, whether increased water exchange frequency and water consumption of CRIS could exacerbate current levels of the agricultural non-point source pollution, causing widespread environmental problems.

To address the research gap, this paper focuses on the analysis of N and P inputs in the CRIS of crayfish breeding, as well as the potential impact of water exchange in the system on the rural environment. In the following section, we introduced the standardized production mode of CRIS in China and its ecological interactions of the integrated system. In Section 3, we introduced the research area, data sources as well as the reason why we chose Qianjiang as the case area. In Section 4, we first introduced the practice of CRIS in Qianjiang and its problems, while at the end of this section we measured the concentration of N and P from several vital points in CRIS. In the discussion section, we discussed the reason for the higher concentrations of N and P in the crayfish production of CRIS, and then, the potential risk for further expanding the non-point source pollution from the perspective of water exchange of CRIS. Finally, the recommendations for controlling the environmental pollution and improvement of CRIS were suggested.

## 2. The Standardized Production Mode of CRIS in China

Crayfish (*Procambarus clarkii*) is a member of the crustacea decapoda family of crayfish which is of considerable economic value in recent years in China. However, during the past several decades, crayfish was regarded as an invasive species or a pest, as crayfish impair young rice plants [25,26], devastate the water drainage systems [27], and so on. Some regions affected severely by crayfish invasion thus tried to use pesticides to eliminate the crayfish. The method is not proving entirely successful in most cases due to the problems of tolerance or toxicity [28]. As a result, many researchers aim to explore a system that integrates crayfish populations and rice fields for leveraging the synergistic effect of ecological cultivation.

Most of these studies and practices can be included in the IAAS, and the most prevalent form of IAAS is rice/fish culture [29]. Generally, rice provides a pleasant external environment for aquacultures, such as lowering water temperature and increasing microorganisms for fish consumption. Fish also help loosen the surface soil, increasing soil permeability and oxygen levels [30]. Nutrients in the soil also degrade faster, making it easier for the rice to absorb. Another contribution of fish is preying on pests and weeds in the field. In addition, their excrement is both the natural fertilizer for rice and soil [31]. In this way, both fish and rice are in an excellent ecological environment, with a virtuous cycle of the circulatory system, enhanced overall function and enhanced productivity [17].

**Figure 1:**
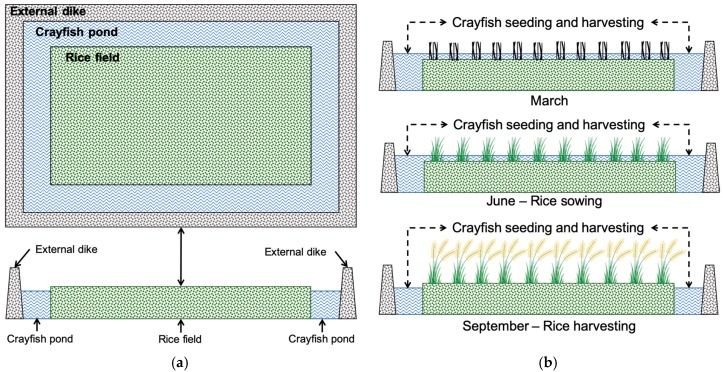
The standardized production mode of crayfish and rice integrated system (CRIS) (**a**) and the production phases of CRIS (**b**).

CRIS continues the pattern of IAAS. According to the FAO Fishery Statistic, two central producer countries of crayfish are the United States (US) and China, where CRIS is most widely practiced [32]. In general, the US usually adopted rice–crayfish rotation. Crayfish can be raised in two basic rotation systems. One is rice–crayfish–rice, and the other is rice–crayfish–fallow. In both strategies, crayfish farming is carried out with the harvest of rice, while the crop stubble can provide food for crayfish. Unlike the US, China usually uses rice–crayfish co-culture that realized the symbiosis between crayfish and rice. The annular crayfish ditch dug along the ridge of the rice field (Figure 1a). During the rice transplanting season, the crayfish seedlings are placed in the crayfish ditch to grow. When the rice seedlings grow strong, then the young crayfish in the ditch led back to the paddy field. In this way, crayfish can be harvested around June and September each year. After September, the winter crayfish seedlings can be harvested the following March again (Figure 1b).

## 3. Data Source and Methods

### 3.1. Data Sources

**Figure 2:**
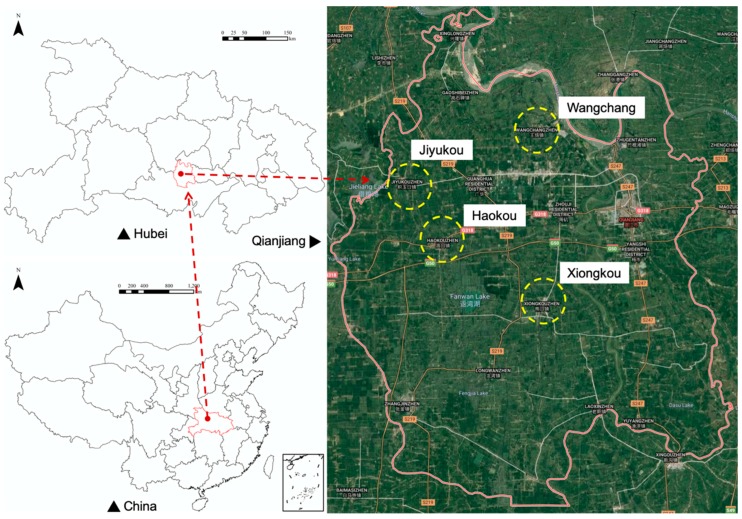
Location of case study.

Qianjiang, Hubei, is located in the Jianghan Plain of China. The practice of CRIS originated in Qianjiang. By the end of 2017, the area of crayfish cultivation in the paddy fields of Qianjiang reached 33,000 hm^2^, the annual processing capacity reached 300,000 tons, and the overall output value of the city’s crayfish industry exceeded 23 billion yuan. According to its practical experience, Qianjiang Bureau of Aquatic Products formulated the *Breeding Technical Regulations of CRIS* and gradually promoted it nationwide. As a result, we chose Jiyukou, Wangchang, Haokou, and Xiongkou, the four towns of a large scale of CRIS in Qianjiang, as the research areas, as seen in Figure 2. We surveyed 280 farmers in August 2018. Eighty percent (n = 224) of them are on household basis in agricultural production. The data we collect includes the area of CRIS, the yield of rice and crayfish per mu, as well as the inputs of pesticides, fertilizers, feed, and so on in the production process over the past three years. In addition to this, we also investigated 20 farmers in the research areas that adopted rice monoculture and collected their input-output data for comparative study.

### 3.2. Methods

As an open agricultural production mode, CRIS frequently exchanges water with the outside environment. As a result, it is hard to accurately calculate the displacement, inflow, and concentration of pollutants in water [33,34]. Therefore, this paper adopted the material balance method to calculate the input and output of N and P in this CRIS [35,36,37,38]. The material balance method considers feed, fertilizer, and agrochemical as the only source of waste in the CRIS. By calculating the N and P content in feed, fertilizer and pesticide input minus the N and P content used in the growth process of rice and crayfish, it is the amount of various pollutants entering the environment. The formula is:

(1) $$K=\left(\overset{n}{\underset{j=1}{\sum }}W_{Bj}\times D_{Bj}+\overset{n}{\underset{j=1}{\sum }}W_{Fj}\times D_{Fj}-W_{Crayfish}\times D_{Crayfish}-W_{Rice}\times D_{Rice}\right)\times 10^{-3}$$

*K* (kg/hm^2^) is the total amount of N or P in a total year for the CRIS. N is the number of terms in the summation formula. Here, *n* represents the sum of N and P inputs for all feeds, fertilizers, and pesticides. $W_{Bj}$ (kg/hm^2^) is the total amount of an individual feed, $D_{Bj}$ (g/kg) is the content of N or P of the feed. $W_{Fj}$ (kg/hm^2^) is the total amount of fertilizers and pesticides, $D_{Fj}$ (g/kg) is the content of N or P in the fertilizers or pesticides. $W_{Crayfish}$ (kg/hm^2^) is the receipt quantity of crayfish, $D_{Crayfish}$ (g/kg) is the content of N or P in the body of crayfish. $W_{Rice}$ (kg/hm^2^) is the total rice harvest, $D_{Rice}$ (g/kg) is the content of N or P in rice. The contents of N and P in feed and fertilizer were mainly determined by the ingredient list. Retained N and P in the crayfish set at 16 g and 0.45 g per kg of its body weight gain respectively [39]. The contents of N and P in rice (including grain, husk, leaf, root and stem) were 22.17 g per kg and 4.88 g per kg, respectively [40].

## 4. Result: The Practice and Input-Output Analysis of CRIS in the Study Areas

According to our investigation, the area of ditches for crayfish production in CRIS accounts for about 15%–20% of the total field area. Table 1 and Table 2 is the average value of material input and output of CRIS and rice monoculture. As indicated in Table 1, CRIS significantly reduced the amount of fertilizers used in the rice production process compared with the rice monoculture. The dosage of N, P fertilizer decreased from 300 kg and 75.5 kg per hectare to 135 and 60 kg per hectare, respectively. Due to the decline in rice area and the use of fertilizers and pesticides, rice production dropped from 9500 kg per hectare to 7400 kg per hectare (Table 2).

**Figure 3:**
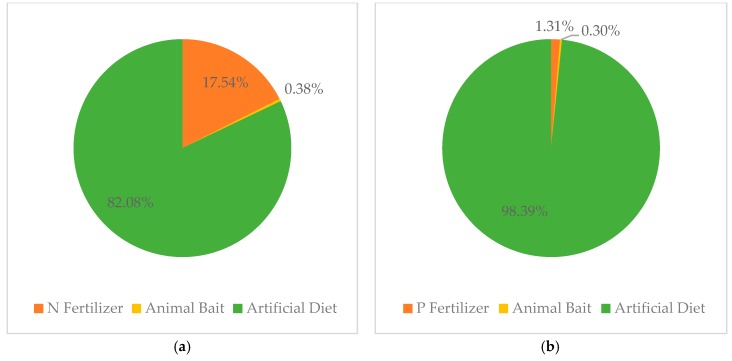
Input ratio of N (**a**) and P (**b**) from different sources in CRIS.

Table 3 is the total number of N and P inputs in the CRIS in one year. In the CRIS, N and P input from fertilizer were 63.05 kg per hectare and 0.9 kg per hectare, respectively. In contrast, the total N and P inputs made by animal bait and artificial diet were 296.45 kg per hectare and 67.90 kg per hectare, respectively. In other words, the feed input required to feed crayfish in CRIS has replaced the fertilizer as the largest source of N and P (Figure 3).

According to the results of material balance method, the output of N and P in CRIS is 76.46 kg per mu and 17.05 kg per mu respectively (Table 4).

## 5. Discussion: The Potential Environmental Risk of the Practice of CIRS in the Case Areas

### 5.1. Profit-Driven Production and the Destruction of Ecological Balance of CRIS

The practice of CRIS in Qianjiang has indeed raised farmers’ incomes and reduced the use of fertilizers. However, this is not due to the ecological benefits of CRIS. Because there is a considerable profit gap between rice and crayfish, and because crayfish production is more straightforward than rice production, farmers are not very active in rice production. Farmers thus increase the area of the crayfish ditch to expand the crayfish production further. Moreover, farmers are generally reluctant to use fertilizers due to the adverse effect on the yield and quality of crayfish. The decrease in rice planting area and the amount of fertilizer per mu have affected the yield of rice. In fact, in order to ensure the stability and security of grain production, the Chinese government has many restrictions on the protection of basic farmland. The *Technical Specification for Integrated Farming of Rice and Aquaculture Animal (TSIFRAA)* require the aquaculture ditch to be less than 10% in an IAAS. However, driven by the enormous profits of crayfish, many farmers have expanded the area to even more than 20% according to our investigation. Under this scenario, though crayfish production has risen, rice production has fallen. As mentioned above (Table 2), the average per mu yield of rice has even been lower than the requirement of *TSIFRAA* of 500 kg per mu.

**Figure 4:**
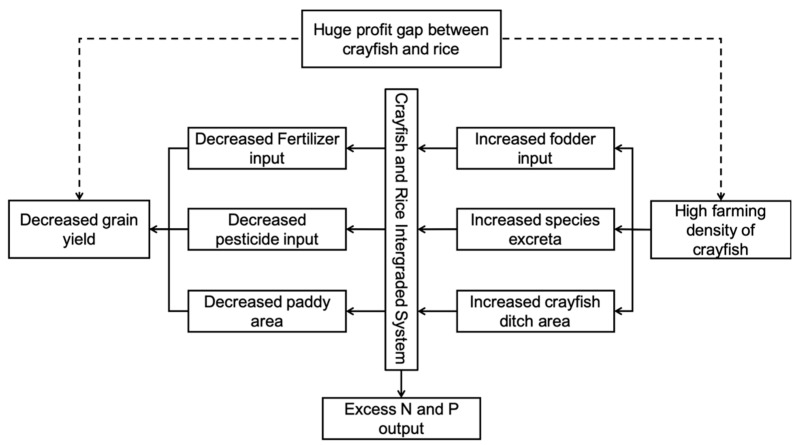
The practice of CRIS in study areas and its ecological consequences.

More importantly, the expansion of crayfish production has upset the balance of ecological exchange in the CRIS. In the CRIS of Qianjiang, because the density of crayfish farming is too high, merely relying on the exchange of substances in the CRIS is not enough to maintain the growth of crayfish. Different from many cases abroad, crayfish and rice production in CRIS of case areas do not have a symbiotic relationship. To ensure the production of crayfish, farmers generally increase the application of feed. According to the investigation, the crayfish in CRIS must be fed every day; otherwise, the crayfish will grow slowly, and the death rate will increase. Additionally, it can be confirmed from another aspect that the crayfish farming density in the case areas is too high. Many crayfish ditches even need to use oxygen pumps to add oxygen to the crayfish. Many studies have proved that in the process of aquaculture, only about 1/3 of the feed nutrients are absorbed by fish or other aquaculture animals, and 2/3 of the nutrients are left in the water [14,29]. As a result, most of the feed nutrients put into aquaculture are released into the environment. The empirical research also confirms this (Figure 4). Therefore, the production process of farmers in the case areas is closer to aquaculture than to CRIS. Rice production remains in CRIS only because of legal and standard restrictions.

### 5.2. Water Exchange of CRIS: The Potential Risk of the New Non-Point Source Pollution

The wastewater produced by aquaculture is also an important source of water pollution that cannot be ignored. Excessive N and P loading into the surface water system are considered to be the most important cause of eutrophication [12,13]. The Ministry of Agriculture of China has set the *Regulation on Quality and Safety Management of Aquaculture* to supervise the discharge of aquaculture wastewater. Although the practice of CRIS in the case area is closer to aquaculture, local governments and farmers still consider it as IAAS. As a result, wastewater discharged in the process of crayfish production is not included in the regulatory system of this regulation.

**Figure 5:**
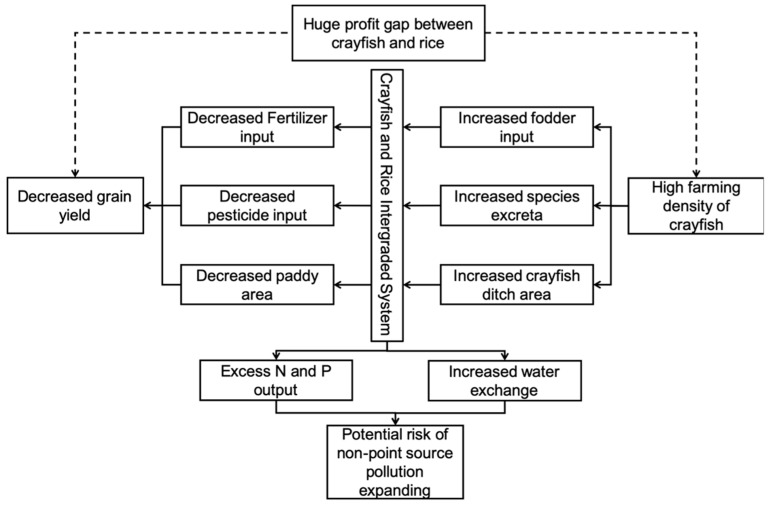
The potential risk of the new non-point source pollution.

As mentioned above, crayfish production has become the primary source of N and P input in CRIS. The results calculated by material balance method ignore the self-purification capacity of the water body and the adsorption of bottom silt. However, from the perspective of environmental security, the extra N and P will increase the pressure on the rural environment. In fact, the local governments in the case area have been aware of this risk. They require some farmers having relatively large farmland (more than 50 mu) to set up a wastewater tank for further treatment. However, according to the investigation, the wastewater is directly discharged into the surrounding water system through the farmland irrigation system. Some farmers are not even aware of the regulation. The increase of water exchange frequency and the generation of new N and P input sources undoubtedly increase the risk of non-point source pollution (Figure 5).

## 6. Conclusions and Policy Suggestions

CRIS practices in China have indeed reduced the amount of fertilizer and pesticides used by farmers. Moreover, crayfish can be harvested three times during a single season of rice planting, which increased farmers’ incomes. However, according to our research, farmers are reducing fertilizer and pesticide use mainly because of concerns about the impact on crayfish production. Moreover, due to the huge profit difference between crayfish and rice, many farmers have expanded the area of aquaculture ditch and increased the quantity and frequency of feed delivery. Rice acreage also decreases accordingly. Therefore, the practice of CRIS in these study areas deviated from the original intention of IAAS and turned out to be an aquaculture. However, since CRIS is not included in the aquaculture regulatory system, N and P, mainly derived from crayfish feed, become the new sources of eutrophication.

Due to the high requirements of crayfish growth on water quality, the frequency of water exchange among farmers in the surveyed areas is very high, especially between June and August. The untreated sewage was discharged directly into the surrounding water system of the study area. Therefore, it can be considered that due to the lack of supervision on the production of CRIS, the crayfish production in the CRIS system has become a new source of eutrophication in the rural area. Moreover, as the irrigation system is directly connected to a broader range of surrounding water systems, there is a risk of further expansion of non-point source pollution. As a result, it is necessary for the local governments to regulate the practice of CRIS, especially limiting the area and density of crayfish farming. In addition, it is urgent to incorporate wastewater from CRIS farming into the supervision system of aquaculture wastewater discharge.

## Figures and Tables

**Table 1:** The input of CRIS and rice monoculture.

Raw	CRIS				Rice Monoculture	
	Rice		Crayfish		Rice	
	Name	Quantity (kg/hm^2^)	Name	Quantity (kg/hm^2^)	Name	Quantity (kg/hm^2^)
Fertilizer (kg/hm^2^)	N	135	-	-	N	300
	P	60	-	-	P	75.5
Feed (kg/hm^2^)	-	-	Animal Bait	50	-	-
	-	-	Artificial Diet	5330	-	-

Note: Pesticides are essential P input sources. However, in the surveyed areas, farmers hardly use pesticides in CRIS.

**Table 2:** The output of CRIS and rice monoculture.

	CRIS		Rice Monoculture
Yield (kg/hm^2^)	Rice	Crayfish	Rice
	7400	2525	9500

**Table 3:** The N and P input of CRIS.

Input	Quantity (kg/hm^2^)	Content of N (g/kg)	Content of P (g/kg)	Application of N (kg/hm^2^)	Application of P (kg/hm^2^)
Rice Seed	37.5	12.38	3.07	0.46	0.12
Crayfish Seed	255	-	-	-	-
N Fertilizer	135	467	-	63.05	-
P Fertilizer	60	-	15	-	0.90
Animal Bait	50	27.50	4.10	1.38	0.21
Artificial Diet	5330	55.36	12.70	295.07	67.69

**Table 4:** The N and P output of CRIS.

	Amount of N (kg/hm^2^)	Amount of P (kg/hm^2^)
Crayfish	177.02	52.12
Rice	−100.56	−35.07
Amount	76.46	17.05
